# Selections and Abstracts

**Published:** 1912-12

**Authors:** 


### SELECTIONS AND ABSTRACTS

### THE TREATMENT OF PULMONARY TUBERCULOSIS BY MEANS OF GRADUATED REST AND EXERCISE

By Oliver Bruce, M. R. C. S., L. R. C. P., London, Eng.

The subject which I have chosen, the treatment of tuberculosis by means of graduated rest and exercise, is one with which few are familiar, and since anything which adds to our knowledge of how to combat pulmonary tuberculosis to the best advantage must be welcome to all, I need offer no excuse for going into the subject in its minutiae.

Probably the bare outlines and general principles of the graduated labor system of treatment are known to most physicians, but an intimate knowledge of the means by which it acts is only arrived at after some experience and is absolutely essential before one can hope to apply it practically with any success. For though not, perhaps an exact science, yet it has an entirely scientific basis and not merely an empirical one.

Before one can obtain this knowledge, however, it is necessary to study to some extent the subject of immunity and the action of bacteria on the blood. In diphtheria we do not make a prognosis of any case on the local condition but far more on the extent to which the diphtherial poison is invading the blood stream. Or again in a pus infection or abscess, although we attend with scrupulous care to the locality of the infection, yet it is only with a view to preventing if possible the invasion of the blood stream by the pus organisms, in other words blood poisoning, and it is on this we base our prognosis, our expectations of the course the disease will pursue. And yet in pulmonary tuberculosis, likewise a bacterial infection, how may physicians rely solely on the local condition and on what they hear through the stethoscope!

You are all doubtless aware that the blood consists of a fluid substance, the plasma, which holds in suspension millions of little cells, which give the fluid its characteristic color, and white cells or leucocytes. It is with the action and properties of these latter cells that we need now concern ourselves. These cells are, so to speak, the scavengers of the body, for the part they play in the metabolism of the tissues is the removal of all noxious substances which may happen to gain access to the body and amongst these are bacteria of all kinds.

Inflammation of any part is due to a flushing of that part with blood which contains a large number of these white cells and the pus which forms consists simply of millions of these little cells for which the poison has been too strong and has destroyed them. They are ameboid in movement, surround the offending object, ingest and destroy it. On account of this action they have been given the name phagocytes and the phenomenon of the destruction of offending substances has been termed phagocytosis.

It was at once evident that an immensely important part was played in disease by these minute cells, especially in bacterial disease. In fact, it was at first thought by Metchnikoff and his school at the Pastur Institute that these cells were the only factor in the destruction of germs that it was merely necessary for the cells to be brought into contact with the germs to take them up and destroy them. But that this idea was an entirely erroneous one was proved beyond question by Sir A. E. Wright in his researches at St. Mary's Hospital, London. There he showed, and his results have been since confirmed by other observers, that a third substance was necessary for the phenomenon of phagocytosis. White cells and bacteria mixed together *in vitro* and incubated give rise to no phagocytosis, but add to them some blood serum and we get the desired effect. If we examine a film of this latter mixture under a microscope we shall find numbers of the bacteria lying within the bodies of the cells whereas in the first instance they all lay outside the cell walls. Now the germs and white cells were common to both experiments, therefore, there is come substance present in blood serum which acts on the white cells in such a way as to enable them to take up and destroy the germs, an action they do not possess of themselves. This chemical substance Wright after many experiments discovered and named opsonin. Carrying his researches still further he was able to demonstrate that the amount of opsonin present in the blood serum of healthy individuals was always the same. For on mixing a definite numbe, of white cells and germs together with one healthy person's serum the number of germs eaten up by the cells would be approximately the same as on mixing the same number of cells and germs with the serum taken from another person in good health. Not so, however, if we substitute the serum of a person suffering from a bacterial infection, such for instance as tuberculosis. We shall then find that the 100 cells have eaten up either a greater or less number of the germs than in the case of the healthy serum, and the ratio of one to the other may be called the opsonic index, an indication as to the amount of this substance opsonin present in a person's blood. Thus if 100 cells under the influence of the healthy serum phagocyte 310 bacteria and under the influence of the infected serum 425 bacteria the opsonic index will be $425/310 = 1.37$ compared with a normal index of 1.00. Obviously then this opsonin is a protective substance which helps the body to eliminate invading germs by means of its action on the white cells. The experiment of the estimation of the opsonic index opens up a very valuable aid to diagnosis, for anyone having an index outside the normal limits should be strongly suspected, at least, of infection. Now, though theorectically speaking, a normal index should always be at 1.00, yet in practice it is found owing to the delicacy of the technique, to vary within certain limits. These have been set at 1.20 and 0.80 and any index outside these figures is considered an abnormal one. At the same time it must be pointed out that in itself one negative result is of little value for at some time or other even infected people's serum will contain a normal quantity of protective substance. Two or more observations should be taken. The first sample of blood should be drawn while the patient is at rest; then some form of exertion should be taken, such as a walk, and another one or two samples of blood drawn some time after the exertion. Then if all three or four samples be within normal limits the assumption is that no disease is present.

Wright discovered during the course of his researches that it was possible to remove all the opsonin for a certain organism from a sample of serum by saturating it with that organism for a considerable time; at the end of that time he found that if he mixed that sample of serum with some white cells and an emulsion of that organism, no phagocytosis took place. If, however, he substituted some other serum he found that phagocytosis did occur, showing that such microorganism has its own specific opsonin. The property of this substance, however, which concerns us most in the study of pulmonary tuberculosis and its treatment by means of graduated labor is that the amount present in the blood serum of an infected person can be made to vary at will by certain means. It is as has been shown a protective substance and makes for health and so it is obviously desirable to raise the quantity present in the serum of all infected people. Wright found that certain things affected the opsonic index and in a specific way. For instance in the case of a gonococcal joint, massage and movement will bring about a strong local reaction accompanied by pain and fever. This reaction moreover is attended by a drop in the opsonic content of the blood below normal. All bacterial diseases are due, not so much to the actual presence of the germs in the tissues but to the fact that the germs throw a poison into the blood stream. But this is not the only action they perform, for this throwing out of toxin, if the dose be not too great, stimulates the tissues to manufacture this antitoxin or opsonin. Thus massage in the case of joint disease, and exertion in the case of a lung lesion cause the germs to throw out a dose of poison, this tends to excite the tissues to manufacture more opsonin and so we get the variations in the index. It is exactly analagous to what happens in vaccination for smallpox or inoculations with tuberculin. As Wright pointed out a certan train of events follows whether it be an artificial inoculation by means of a syringe or an artificial autoinoculation by massage or exercise. Immediately following the administration of the dose he observed a drop in the opsonic content of the blood and this he termed the negative phase. At a greater or lesser interval according to the size of the dose, a positive phase followed during which the opsonic index was above the normal. This in time gave place to what he termed a phase of maintained high level during which the index returned nearly to the point whence it started.

Graduated labor is a service of artificial autoinoculations. Now a patient with advanced disease, showing a high temperature every evening in spite of being at rest in bed is undergoing a series of excessive autoinoculations accompanied by a fluctuating opsonic index. This means that the dose of poison thrown out by his germs into the blood stream is so great that an insufficient amount of protective substance is formed. If the opsonic index of such a patient be examined at certain intervals over a period of 24 hours it will be found that when his temperature is high his opsonic is low and vice versa. The opsonic index in other words varies inversely with the temperature. This is a most important point which was brought to light in the course of over 300 blood examinations performed by Dr. A. C. Inman and myself at the Brompton Hospital. For in regulating the amount of exercise to be prescribed in graduated labor, some guide is necessary, some indication as to the amount of protective substances which are being elaborated as the result of the exercise. It would be found far too laborious a task to have to take the opsonic index as a guide and this knowledge enables us to substitute for it the temperature chart. The clinical symptoms and feelings of the patient, however, must be very carefully studied at the same time.

There has been much controversy as to the reliability of the opsonic index but, in a paper such as this I cannot, of course, go into so vexed a question. I should like, however, to describe a certain experiment which more than anything else impressed me with its value. When Dr. Inman and I started our work at the Brompton Hospital the physicians were very sceptical about it and tried in every way they could to trap us. They sent us for examination amongst other samples of blood those of three cases ot bronchiectasis in which no tubercle bacilli had been found by the house physicians. We estimated their opsonic indices and made two of them abnormal to tubercle and one normal. We then proceeded to examine the sputa in each case by carbolizing and centrifugalizing it and by these means were able to demonstrate the undoubted presence of the tubercle bacilli in the two abnormal cases, finding none, however, in the normal case.

We have then two main facts to work on, that the amount of opsonin or antibody in the blood can be raised by exercise and that the temperature chart and feelings of the patient are a sufficient guide to an experienced observer as to whether the amount of exercise prescribed is too great or too little.

Paterson has fixed the danger mark, the point signifying excessive autoinoculation, at 99° F. in the case of men and 99.6° F. in the case of women. Our first object therefore in every case is to reduce the temperature to below these points and this, of course, we do by rest in bed. Ordinary rest in bed will usually attain this object, but it is an interesting fact discovered by Paterson and Inman, that if what is known as "absolute rest" is prescribed the temperature will return to normal more quickly still. By absolute rest is meant the treatment prescribed in typhoid fever where the patient is not allowed to move for any purpose whatever. Now when this point has been reached and the patient's temperature has been continuously below the normal for ten days we know that excessive autoinoculation has been checked and that this output of antibodies is sufficient and that his output of antibodies is sufficient to cope with his poisons. It is at this point that other methods of treatment stop short. For if nothing more than rest and forced feeding be prescribed there will be no stimulation of the tissues to form antibodies in excess of poisons. This point too was clearly shown during the course of our investigations. We examined the blood of many patients while at rest or an unchecked walking exercise at the Brompton Hospital and again the same patients while on strict graduated labor at the sanatorium. In every case on the former treatment the opsonic indices were low, and in every case the latter treatment raised them high above normal. This is why graduated labor is so far superior to other methods. Our object then is to raise the patient's opsonic index above the normal line and it has been shown that this can be done by means of exertion. The exertion, however, must not be too great or the output of toxins will swamp the tissues and an exactly opposite result to that which we desire will occur.

It is my practice to prescribe as the first step, an hour a day out of bed, sitting in a chair. If no rise of temperature occurs, on the third day I allow the patient to dress himself for the hour he is up, as the act of dressing after a long period in bed is a trying one. Still keeping a careful watch on the temperature chart and feelings of the patient I gradually increase the number of hours spent out of bed to two, four, six, eight and ten hours with an interval of two days between each increase, until finally the patient is getting up for the whole day, though doing no more than rest in a chair. If from time to time we examine such a patient's opsonic index, we will find it is tending to rise, but not to any very great extent. We are gradually immunizing him to larger doses of poison but we want to carry it further still and raise his opsonic content as high as we possibly can. This we can only do by giving him bigger doses of poison and so the next step is to order him walking exercise which must be very carefully graduated or the dose will be too great. I start my patient at half a mile a day, walking quite slowly and preferably over gently undulating ground. The distance is gradually increased until finally he is walking six miles a day.

The patient should lie down for an hour after each period of exercise and this "rest hour" should be as vigorously enforced as is the exercise, for it is then that the positive phase should take place and the protective substances be elaborated. Any rise of temperature to the "danger mark" or over indicates excessive exertion and should be dealt with accordingly. The point has now been reached when the patient is walking six miles a day without any rise of temperature. During all this time, however, he has only been using the muscles of his lower limbs and as the muscles of his arms and trunk have more effect on the expansion of his lungs, some form of exercise which will bring these into play should be the next step. It is from this point that the real "labor" part of the system begins and I think I cannot do better than tabulate shortly the various grades as performed at the Brompton Hospital Sannatorium. In all the grades the work is carried on for four hours a day.

*Grade I.*—Small baskets. A weight of about 10 pounds of mould or other material is collected into baskets and carried a distance of about fifty yards, total weight carried about 8½ cwt.; distance traveled about 7 miles. For this may be substituted such tasks as light weeding, potting, picking out seedlings, picking off dead flowers, watering plants and painting. Time spent on this grade one week.

*Grade II.*—*Larger baskets.*—A weight of about 18 pounds is carried a distance of about 50 yards; total weight carried about 15 cwt.; distance traveled about 7 miles. For this may be substituted weeding with hand fork, planting out in open ground, cutting vegetables and watering plants. Time spent on this grade one week.

*Grade III.*—Sweeping paths and grass, cutting grass edges, chopping firewood, hoeing, painting with large brush and cleaning windows. Time spent on this grade one week.

*Grade IV.*—Using a small shovel or digging with a small fork. Five men can pull a hand cart containing soil or stones, moving grass or rolling. Time spent on this grade two weeks.

*Grade V.*—Using large shovel or pick axe or digging with large fork. Pulling down trees and trenching ground 3 feet deep, hauling stones, etc., in cart, using wheelbarrow and doing general heavy navvy work. Sawing and planing. Time spent on this grade three weeks.

*Grade VI.*—Similar to Grade V, patients working for six hours a day instead of four.

The work for the women should be slightly modified. Small baskets should carry a weight of 8 pounds and large baskets 15 pounds. Besides this they can sweep paths, do light gardening, clean out small chicken houses and prepare food for the chickens. Their final grade should consist in scrubbing floors and such heavy work as they may have to perform at home.

Naturally such a routine as I have sketched here will be found too ambitious for a small sanatorium containing but 30 or 40 patients. Moreover the different conditions which prevail in

Canada, especially in the winter months, will necessitate a modification of this routine and some difficulty will be experienced owing to the land being snowbound. Much of this difficulty will disappear, however, if one considers that any work will serve the same purpose provided the amount of energy expended be proportionate.

At the Byron Sanatorium during the winter months I instituted the following forms of work: Collecting small wood into heaps for kindling purposes, sawing trees into lengths with a cross-cut saw for burning in the various furnaces, and shoveling snow. Now more outdoor work is possible and the men are employed in various forms of gardening, such as cutting out and digging up new flower beds and manuring them, hoeing, weeding and raking, also painting gates, fly screens, etc. The women in removing stones from the ploughed land, weeding and hoeing. Dumbbell exercises and skipping too have proved a very useful form of exercise for both sexes as an adjunct to outdoor work.

The patient should be gradually immunized to harder forms of labor until finally he is able to work 6 hours a day at the most strenuous exercise that can be found for him without any rise of temperature. At the same time the work, if possible, should always have some aim and object apart from its curative function or it will become uninteresting and tedious.

When Paterson originated the graduated labor system of treatment he had no knowledge of the scientific basis which was the real cause of its success. Wright's epoch-making work had not then been given to the world and the action of bacteria on the blood was but very imperfectly understood. Paterson instituted it from merely common sense motives, for he said clearly that previous methods did not go far enough towards raising the resistance of patients and making them fit for work. It would be necessary for all his patients to return to work at the conclusion of their treatment and he knew that after a regime of rest and milk they would very soon break down again. His idea, therefore, was to raise their muscles to such a high state of resistence that they would be able to perform the hardest work without detriment. He had noticed that many of his cases had been working hard right up to the day of admission to the sanatorium without harm. "If consumptive persons under adverse circumstances and without any medical guidance could act in such a way, ought they not to be able, under ideal conditions and with the work carefully graduated to suit their physical condition, to perform useful labor?" In these words he sums up his reasons for the great change he intended to make in the treatment of the consumptive. A few years later Wright published his work and Inman's investigations put the whole system on a thoroughly scientific footing.

Our examinations of the bloods of the Frimley patients went to prove that all Dr. Paterson's conclusions and theories were correct. Were we to ask him for the blood of a patient with a high opsonic index he was able to pick one out, or if that of a man with a low index, again such a patient was found and invariably he was right. From this it can be seen that though the opsonic index is the best guide to the amount of protective substances each patient is elaborating in response to the exercise, yet with experience the temperature chart and clinical symptoms of the individual can be made to take its place. This scientific knowledge however has been of inestimable value in another direction as well. Up to this time Dr. Paterson had been obliged to work very carefully in increasing each patient's exercise for fear of an overdose. These fears were then proved groundless and as a result the same number of beds can now accommodate 40 per cent. more patients in the year than when graduated labor had to stand on its own merits with no scientific proof at its back.

*The blood examinations offered convincing proof that the work actually did result in autoinoculations with the patient's own antibacterial products.*

It was a striking feature of these investigations the very high indices the majority of the patients showed as the result of the labor and moreover it was noticed that the harder the work performed the higher rose the index.

There were however, some patients who were apparently marking time. Although in good condition they did not appear to be deriving the full amount of benefit from the particular grade of work they were on and in some cases even they had occasional rises of temperature. Dr. Paterson thought that perhaps what this type of patient needed was still harder work. Accordingly they were promoted to a yet higher grade with a successful result and when we investigated the bloods of these patients the reason for this was made manifest. The first grade of work had not been sufficient to excite a satisfactory stimulus on the part of the tissues to manufacture antibodies and their indices were low. On putting them onto the higher grade, however, the resulting stimulus was sufficient. There was a corresponding increase in the amount of opsonin formed and a rise in the index. This rule has in consequence been followed ever since with success.

The effect of over-exertion was well shown in the case of several patients who on account of feeling so much benefit from the work become over-zealous and did too much against orders. These patients all had rises of temperature accompanied by a drop in the opsonic index as revealed by blood examinations. The same thing was recorded in the case of a musical patient who played the piano before he was in a fit state to do so. His temperature chart showed a rise to 101 degrees and his index a drop to much below normal. This latter case emphasized the fact that rest is just as important as the work and should be rigorously enforced.

The next fact that was revealed by these investigations, perhaps the most interesting of any, was that whereas patients on the higher grades of work showed very high indices, the same patients when on the final grade and ready to be discharged had indicies which never varied from the normal, however, hard the work. The reason for this is that when the final grade is reached their lesion is so shut off from the blood stream by fibrous tissue that no toxins can be poured into the circulation and consequently there is no need for the formation of antibodies above the normal amount. Such patients are in the true sense of the word cured and may be discharged with confidence. It would be an ideal state of affairs if at the conclusion of the treatment of every consumptive person a series of indices could be taken to determine if the hardest work was having any effect at all on his antibacterial output or not. If any of the indices proved to be abnormal, then further treatment would be indicated, but if not, then the patient might return to his occupation without fear.

After the Brompton Hospital Sanatorium had been opened some 18 months and further experience gained, it was seen that many patents who had a rise of temperature to 99 degress F. as the result of over-exertion, were not only none the worse for it, but in most cases actually better. This is explained by the fact that though the over-exertion had produced such a dose of toxin as to cause a marked negative phase, yet the response on the part of the tissues had been correspondingly great in the formation of opsonin and so good had resulted instead of harm. Such cases, however, cannot be too carefully watched and if headache or malaise accompany the rise of temperature, stringent measures must be taken. If on the other hand, the rise of temperature is the only indication of over-exertion, after a few days walking exercise, the patient may be allowed to resume work in the grade in which he suffered from the temporary setback. Some patients even go so far as to say that they date their real improvement from one of these slightly excessive auto-inoculations.

There is, however, another side to the graduated labor system, and that is the mental and moral effect produced by the work. Patients are apt at first to be sullen and discontented when made to work, but as soon as they feel the great benefit which accrues their demeanor completely changes and they take to it with great zeal. For this reason it is often advisable to give them some occupation while they are confined to bed unless they are on "absolute" rest, for it prevents them from continually brooding over their ailment. As a certain eminent physician once put it, "no *fool* was ever cured of consumption," and it is a fact that the more determined a patient is in his own mind to get well the better will his progress be.

The conditions prevailing at the Brompton Hospital Sanatorium are obviously very different to those in any similar institution in Canada. To begin with it has its own hospital containing some 300 patients at its back, and therefore only the very best cases are picked out for treatment at the sanatorium. More important still is the fact that all patients, early and advanced cases alike, can be watched at the hospital, and any that improve can be selected for graduated labor treatment.

A sanatorium not having a hospital at its back must refuse all advanced cases, and yet we know that many of them could be cured if given the chance. In these days the extent of the disease is not so important a factor as it was considered to be in in times past, for it is recognized that a man may have an advanced lesion extending over some years and at the same time may have a good resistance. Such a man will do better than one with a small and early lesion, but a poor resistance. There is, we know, a percentage of early cases, though happily a small one, on which no treatment will have any effect, however early it be instituted. There are probably two factors bringing about this state of affairs, a lowered resistance and a virulent type of organism. A hospital acts as a siftng ground for all these different types of cases, whereas a sanatorium acting by itself has to do its own sifting and it is impossible for the medical officers' pronosis to be always correct.

The patients at the Brompton Hospital Sanatorium number 125 and this, added to the fact that there are very few patients confined to bed, enables them to undertake more ambitious work than can be done in most sanatoria in Canada. For example, the patients have completed, entirely by their own labor, a reservoir capable of holding 500,000 gallons of water. For this purpose they cleared the land of trees, excavated it, mixed all the concrete and laid all the blocks of gigantic. A skilled engineer is kept on the staff for the special purpose of instructing them in the work and under his guidance they are now engaged in building an open air chapel and a Dutch garden.

It is found then that rules and regulations have to be most vigorously enforced. The patients are not allowed to abstain from work on account of inclement weather or indeed on any pretext whatever, save that of illness, for Dr. Paterson very rightly thinks that to work one day and not the next is harmful and opposed to the success of the system. Certainly it is a striking fact that despite periodical soakings the patients at Frimley are remarkably immune to colds and pneumonia and yet they may be seen working in all weathers.

A system of punishment for infringment of orders has been instituted. Passes are issued to patients on certain of the higher grades which permit them to take walks in the country instead of doing their ordinary work on one or two afternoons a week. If however, any trouble is experienced in making them perform their alloted tasks, their next pass is suspened and as the privilege of these outings is very much appreciated, it is seldom that the punishment has to be repeated. In order that the system may be successful it is absolutely necessary to have in force very stringent rules and regulations though they need not be quite so rigorous in this country as in England where the class of patient is somewhat different.

It should be explained to every patient on admission in what the system consists and that he will be ordered a certain amount of rest and a certain amount of work, and that this is now more important than any medicine, and is in itself a form of treatment and nothing else. Further, it should be pointed out that as he himself will not know how much exercise should be taken at any time, he must be guided absolutely by the medical officers' orders, for while too much exercise will result in harm, too little will not secure the amount of benefit required.

At the Byron Sanatorium I also give the patients short lectures at intervals, explaining how the system works, and I find that it gives them a greater interest in their cure, besides making them see it is for their own benefit and not for the benefit of the institution.

To sum up, the graduated labor system of treatment is an effort at the scientific cure of the consumptive. It aims at raising the amount of antibacterial substance in his blood to as high a degree as possible and keeping it there by gradually increasing doses of his own tuberculin.—*American Medicine, Nov.* 1912.

### SURGERY OF NEPHRITIS

The surgical treatment of nephritis, independent of stone, tumor, urethral obstruction, or tuberculosis infection, has been evolved in part, at least, from operative measures undertaken because of a mstaken clinical diagnosis. After splitting a panful and bleeding kidney for either suspected stone or other gross lesion, it was noted not once but many times that though the diagnosis was proven wrong and simply a nephrotomy had been done, the patient who recovered from the operation was relieved of his symptoms and remained, in some cases at least, definitely and permanently well. A further impetus to surgical procedure in the absence of gross and demonstrable lesions was given by Edebohls' contribution on the subject of decapsulation for the relief of anuria and for the continued betterment of renal elimination in cases of advanced Bright's disease.

Theoretically descapsulation was based on the belief that it accomplished an immediate relief of tension, by splitting and turning back the capsule, and by the hemorrhage incident to this operation; and that subsequently a collateral circulation was formed which would provide the kidney with a sufficient quantity of constantly changing blood for the proper performance of its function.

Both clinical and experimental study have shown that an adequate collateral circulation cannot be expected to form; that in place of a stripped capsule there is formed a dense fibrofatty investment with little vascularity and capable of producing more pronounced pressure than the thin proper capsule of the kidney. None the less there are many recorded cases which apparently show the immediate benefit to be derived from decapsulation where there is pronounced urinary intoxication. Since, however, Bright's disease has for one of its characteristics extraordinary and apparently causeless fluctuations in symptomatology, those apparently moribund recovering at times under almost any treatment, it has been held that the evidence for decapsulation is still far from convincing.

The subject of surgical intervention for the relief of symptoms incident to nephritis has been for many years one of major interest to Kummell, who contributes the results of an admirable clinical study (*Archiv fur Klinische Chirurgie*, 98 Bd., 3 Heft). Considering first acute nephritis, Kummell regards that incident to infectious processes, and particularly to scarlet fever, as accompanied by a greatly increased intrarenal pressure, and quotes Harrison to the effect that this pressure is comparable to that of the eye in glaucoma, the kidney affection indeed being called pernicious renal glaucoma. The intrarenal pressure first functionally and then organically destroys the renal parenchyma, causing either death or invalidism from chronic nephritis. The indication for either splitting the capsule or nephtrotomy would seem to be fairly plain in cases of this nature accompanied by anuria.

Toxic nephritis caused, for instance, by sublimate, carbolic acid chloride of lime, or other chemical poisons, seems also to call for direct surgical intervention where anuria results from it. The author records a striking case in which no urine was passed for seven days. A few hours after decapsulation 500 gramme. were passed, and in eighteen hours over a liter in all. Doubtless the kidneys would have continued secreting had not the patient died.

As for that form of acute nephritis characterized because of its major symptom as eclampsia, more than 100 cases have been reported. Sippel notes that of 46 decapsulated eclamptics 30 recovered. Poten has recorded 98 operations with 38 deaths.

Acute infectious nephritis, by which term is meant that of hematogenous origin, characterized by multiple abscesses, is considered particularly amenable to surgical treatment. The condition is characterized by the general symptoms of violent infection; blood, pus, and bacteria in the urine; rigidity of the muscles over the kidney suggesting a peritonitis; and in its localization as to the kidney affected by urethral catheterization. Kummell reports twenty-eight cases varying in age from sixteen to sixty years; of these three died. All of the cases were unilateral; colon bacilli, stapylococci, and strepococci were the infecting organisms. Seventeen nephrectomies and eleven nephrotomies were performed. Kummell announced himself as now less inclined to nephrectomy than in former days. This operation is followewed by prompt healing, but in spite of all modern methods of examination the surgeon cannot be absolutely assured of the complete health of the remaining organ. Two of his three mortalities occurred from anuria coming on more than a week after an apparently entirely successful operation. Nephrotomy is slow to heal, sometimes bleeds, and sometimes leaves a long standing fistula. It, however, is the operation of choice unless the kidney is obviously so completely destroyed as to leav no secreting substance.

As to the question of spontaneous healing of acute hematogenous nephritis, it can scarcely be doubted that such can occur, though this result must be an exceptional one, so exceptional that it should have no weight in considering the question of operation; this should be undertaken even when the affection is demonstrated to be bilateral. In unilaternal infection sepis is always violent, and this in itself may cause a toxic nephritis in the kidney not yet bacterially involved. It therefore follows that the infected kidney should be treated by incision or complete splitting as soon as the diagnonis is made. Indeed, Kummell regards acute infectious nephritis as preeminently a form of inflammation which calls for operative interference and responds favorably to this when it is performed in time.

As to chronic nephritis Kummell first considers the affection which he calls nephritis dolorosa, formerly termed nephralgia or renal neuralgia. These cases were originally cut for stone or other gross lesion. Later it was observel that the capsule was thickened and adherent to the fatty envelope. There was fibrous infiltration of the kidney itself and hyperemia of the parenchyma. Portions of the kidney substance removed at operation showed chronic nephritis, as did the urine obtained by ureteral catheterization. Always it was observed that there was a circumscribed nephritic process or one in its early stages. Even at the present day a differential diagnosis from a small uric acid calculus may be difficult or impossible. This may be acute colicky attacks or persistent pain with exacerbations usually located in one side. It is undoubtedly true that chronic nephritis is in the vast majority of cases a bilateral process, none the less it has been abundantly proven by clinical reports that exceptionally it may be limited to one side. Even in those cases of unilateral nephralgia a careful investigation usually shows involvement of both kidneys. Kummell has seen 13 cases in which the most pronounced symptom was unilateral pain, and in 10 of these a diagnosis of nephritis could be made before operation. In four cases decapsulation was practiced, in nine the kidney was split. One of the patients was operated on in 1891. He suffered from left-sided colic, which was supposed to be due to stone. Nephotomy completely relieved him of his pain. He later died of nephritis. All the other patients were completely cured, 3 for six years, 2 for four years, 2 for three years; the rest were operated on within the last year. This experience is corroborated by many reported cases and apparently justifies the operation of decapsulation or nephrotomy in these cases.

Hemmorrhagic nephritis, variously called renal hemophilia, renal expistaxis, and hematuric nephralgia, the latter name because it is often accompanied by pain, was at one time characterized simply be case reports without pathological findings; later it was recognized as a form of chronic nephritis. The distinction from tumor is by no means an easy one to make. The catheterization usually shows a healthy bladder and bleeding from one kidney which may be extremely free, and may, by occluding the ureter, cause renal colic, followed by expulsion or typical, worm-like casts. Traces of albumin and leucocytes are usually observed in the urine, but the casts are as a rule not found. The patients are generally cachectic, and indeed a single suggestive symptom in regard to the nature of the affection may be the demonstration of a bilateral nephritis. Operative treatment is the only therapeutic measure which has proven of service. Indeed intervention is called for from the diagnosis standpoint alone, since if the bleeding be due to tumor its early and complete removal offers the only chance of cure. Examination of the extirpated kidneys showed an early glomerular nephritis with such slight alterations of the renal tissues that only serial sections of the entire kidney can demonstrate it beyond controversy. Macroscopically the kidneys were apparently entirely normal; the same findings resulted from the examination of small excised portions.

Kummell has observed 1 cases of hemorrhagic nephritis, always, in so far as the bleeding was concerned, unilateral. One patient, a profoundly anemic man, sixty-two years old, died of hemorrhage during operation. Autospsy showed a chronic bilateral nephritis. One young man died eight months after nephrectomy from uremina incident to progressive degeneration of his remaining kidney. One patient on leaving the hospital exhibited a low freezing-point with casts and albumin in his urine. The remainder were cured. Many of them had been observed for several years. Nephrectomy was performed nine times, decapsulation four times. Kummell favors decapsulation. Should this fail nephrotomy should then be practiced. It is obvious that these cases of renal neuralgia can by decapsulation or nephrotomy return to work with apparently perfect health, even though from the strict anatomical standpoint a cure cannot be expected.

As to medical nephritis, or Bright's disease, by this is meant the usual parenchymatous or interstitial nephritis characterized by albumin and casts in the urine, nearly always bilateral, and accompanied by edema and marked deterioration in health. Kummell has operated on 26 cases. Two died within a few days, one in two months, one in five months, one in a year and a half, and one in six years. In five cases the operation was without benefit. In 14 there was more or less distinct improvement. In none of the patients were albumin and casts entirely wanting after operation, with one exception. A young man twenty years old suffered from nephritis for four years. The year after double decapsulation he was perfectly well and the urine was normal. Lessening the quantity of albumin and of casts, general improvement in health and gain in weight and the ability to resume work have been observed in ten cases. In the most severe cases with uremia and anuria at least a transitory betterment may be expected, nor if it be done under local anestesia, if this is possible, is the operation a difficult or dangerous one. No death is reported as the direct consequence of surgical intervention. Doubtless nephrotomy would be more a radical and serviceable procedure than decapsulation, but Kummell believes that this operation is too formidable and attended by too great a bleeding to justify it. As to the comparative merits of nephrotomy and decapsulation, the former seems the more efficient in accomplishing relief of renal tension.—*Therapeutic Gazette.*

